# The Core Pattern Analysis on Chinese Herbal Medicine for Sjögren's syndrome: A Nationwide Population-Based Study

**DOI:** 10.1038/srep09541

**Published:** 2015-04-29

**Authors:** Ching-Mao Chang, Hsueh-Ting Chu, Yau-Huei Wei, Fang-Pey Chen, Shengwen Wang, Po-Chang Wu, Hung-Rong Yen, Tzeng-Ji Chen, Hen-Hong Chang

**Affiliations:** 1 Center for Traditional Medicine, Taipei Veterans General Hospital, Taipei, Taiwan; 2 Graduate Institute of Clinical Medicine, and Graduate Institute of Traditional Chinese Medicine, College of Medicine, Chang Gung University, Taoyuan, Taiwan; 3 Department of Computer Science and Information Engineering, Asia University, Taichung, Taiwan; 4 Department of Medical Research, China Medical University Hospital, China Medical University, Taichung, Taiwan; 5 Department of Biochemistry and Molecular Biology, School of Life Sciences, National Yang-Ming University, Taipei, Taiwan; 6 Department of Medicine, Mackay Medical College, New Taipei, Taiwan; 7 Cloud Computing and System Integration Division, National Center for High-Performance Computing, Taichung, Taiwan; 8 Division of Rheumatology and Immunology and Department of Education, China Medical University Hospital, Taichung, Taiwan; 9 Research Center for Chinese Medicine and Acupuncture, and School of Chinese Medicine, China Medical University, Taichung, Taiwan; 10 Department of Family Medicine, Taipei Veterans General Hospital, Taipei, Taiwan; 11 Institute of Hospital and Health Care Administration, School of Medicine, National Yang-Ming University, Taipei, Taiwan

## Abstract

This large-scale survey aimed to evaluate frequencies and patterns of Chinese herbal medicine (CHM) used for Sjögren's syndrome (SS) in Taiwan by analyzing the National Health Insurance Research Database (NHIRD) for cases in which CHM was used as an alternative therapy to Western medicine for improving patients' discomforts. We analyzed cases of SS principal diagnosis (ICD-9:710.2) with a catastrophic illness certificate (CIC) in traditional Chinese medicine (TCM) outpatient clinics from three cohorts of the Longitudinal Health Insurance Database (LHID) in the NHIRD between 2002 and 2011. CHM prescription patterns for SS were evaluated from claimed visitation files and corresponding prescription files. There were 15,914 SS patients with CIC (SS/CIC), and we found only 130 SS/CIC cases visiting TCM clinics in LHID2000, 133 in LHID2005, and 126 in LHID2010. After removing duplicate data, 366 SS/CIC and 4,867 visits were analyzed. The 50–59 year age group showed the highest ratio (29.51%) in both women and men. “Qi-Ju-Di-Huang-Wan” and “Xuan-Shen” (*Scrophularia ningpoensis* Hemsl.) was the most commonly used formula and single herb, respectively. “Qi-Ju-Di-Huang-Wan, Gan-Lu-Yin, Xuan-Shen, Mai-Men-Dong (*Ophiopogon japonicus* (L. f.) Ker-Gawl.), and Sheng-Di-Huang (raw *Rehmannia glutinosa* Libosch)” were the core pattern prescriptions in treating SS/CIC.

Sjögren's syndrome (SS) is an autoimmune disease that mainly involves exocrine glands such as lacrimal and salivary glands. It causes glandular dysfunction, manifesting in dry mouth and dry eye^1^. SS can be classified as either primary or secondary^2^. Primary SS (pSS) presents as SS alone, whereas secondary SS occurs when another autoimmune disease, such as rheumatoid arthritis (RA), systemic lupus erythematosus (SLE), or systemic sclerosis, is present. In Taiwan, the incidence of pSS from 2005 to 2007 was 6.0 (per 100,000 inhabitants) with a female/male ratio of 9.9^3^. The prevalence of SS was ranked third among autoimmune rheumatic diseases in Taiwan from 2000 to 2008^4^.

The pathogenesis of pSS remains unclear, but some clues support the hypothesis that the dysfunction of B cells and T cells leads to autoimmune epithelitis^5^. In addition, type I interferon and B cell-activating factor play important roles in the pathogenesis of pSS^6^.

Because some individuals with SS have a high risk of developing non-Hodgkin's lymphoma^7^, and some SS patients treated with conventional Western medicine still have discomforts, such as pain and fatigue^8^, these individuals have sought complementary and alternative medicine (CAM) for relieving symptoms.

Chinese herbal medicine (CHM)^9^ and acupuncture^10^ are common CAM therapies widely accepted by SS patients. Some randomized control trial have been performed in treating SS, but no specific CHM could be recommended for treating SS^11^. However, most of these trials suffered from poor methodological quality of trials and high heterogeneity of interventions. Hence, no specific CHM has been recommended for treating SS.

In Taiwan, National Health Insurance (NHI) has covered both Western medicine and traditional Chinese medicine (TCM) medical care since 1995^12^. Most of the general population (98%) in Taiwan was covered in the NHI program at the end of 2012. A recent large-scale investigation about TCM usage for SS in Taiwan showed that Qi-Ju-Di-Huang-Wan was the most commonly prescribed Chinese herbal formula for SS^13^. However, the report included only one LHID cohort (1 million randomly sampled subjects), and the results may have been overestimated among patients recruited with regard to TCM visits and the average daily dose of Chinese formula. Hence, we conducted a national population-based study from the three cohorts of LHID in Taiwan for CHM usage among SS patients from 2002 to 2011. We focused on SS patients with CIC and SS as the principal diagnosis visiting for TCM.

## Methods

### Data Sources

The NHI program has a unique database that was implemented in Taiwan in 1995 and covers almost the entire population. Accordingly, TCM outpatient service for adults and children was analyzed from that time^14^,^15^. Nationwide medical information is recorded as electronic claim data in the National Health Insurance Research Database (NHIRD), which contains gender, age, dates of encounters, disease diagnosis of patients, and prescriptions for all beneficiaries^16^. Scrambling cryptogram was performed on these data to protect patient and institutional privacy. The study population was defined by disease diagnosis according to the International Classification of Diseases, Ninth Revision, Clinical Modification (ICD-9-CM). As we conducted a retrospective analysis of the NHIRD and all the individual information data are de-identified, we could not obtain informed consent from included patients. This study was approved by the Institutional Review Board of Taipei Veterans General Hospital (VGHIRB-2013-04-005E).

### Identification of Patients with Sjögren's Syndrome

We designed a national population-based study to analyze three cohorts of 1 million random sample subjects selected from all beneficiaries of the NHI program in Taiwan, and we surveyed the CHM usage rate and patterns to determine the utilization of subjects with the principal diagnosis of SS treated in TCM outpatient clinics. The usage of principal diagnosis of SS in TCM visits can reduce measurement bias that would be imposed by TCM visits for non-SS treatments.

**Figure 1:**
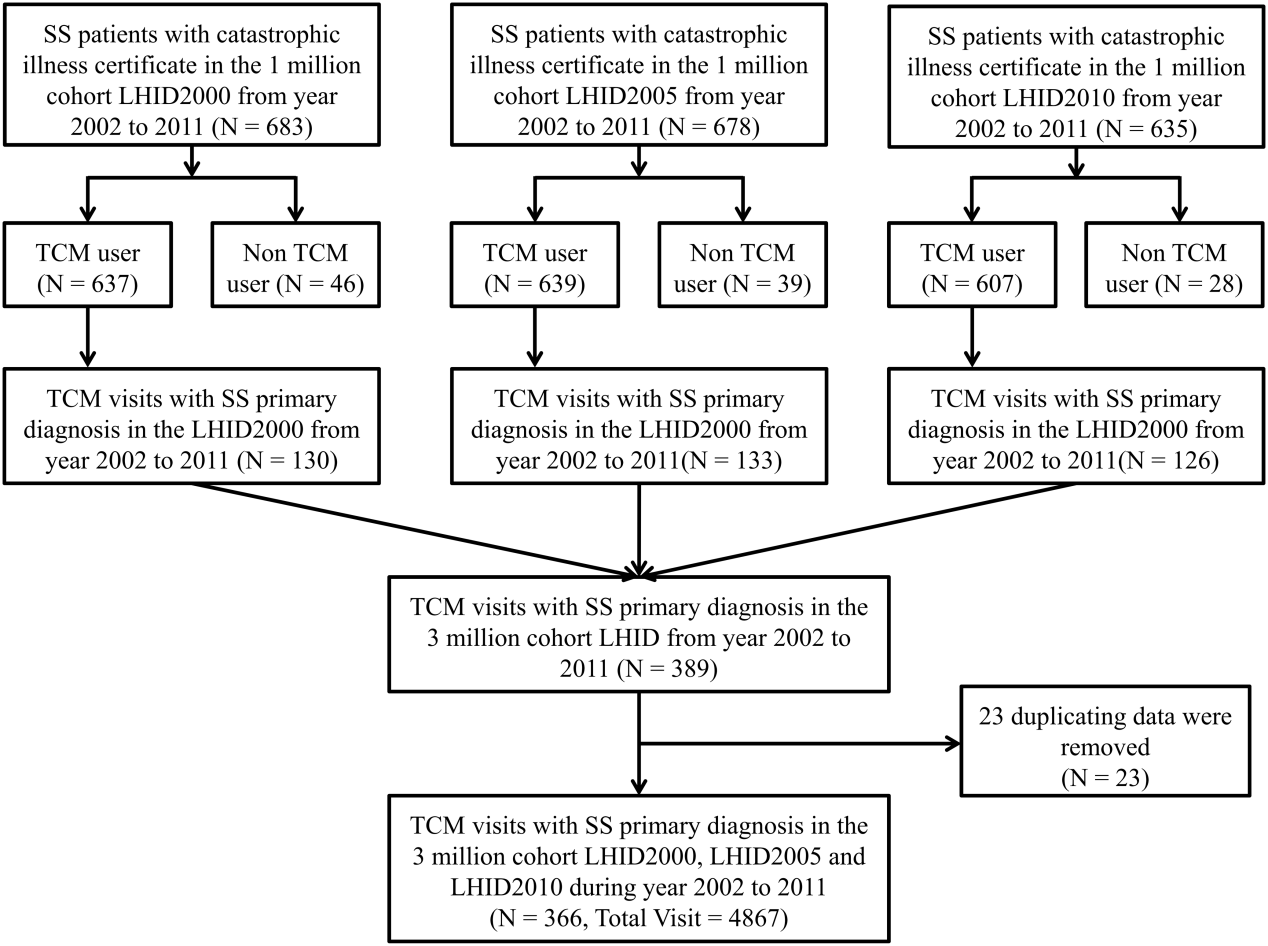
Flowchart of recruitment of patients with Sjögren's syndrome and a catastrophic illness certificate from the 3 million random samples. We identified Sjögren's syndrome in individuals with an ICD-9 code of “710.2” and a catastrophic illness certificate in Taiwan between 2002 and 2011, and they were matched with three cohorts of 1 million random samples from the LHID2000, LHID2005, and LHID2010. Subjects with a catastrophic illness certificate were included after duplicate data were removed.

SS is one of the 31 categories of major illnesses or injuries that result in a patient holding a catastrophic illness certificate (CIC). As SS and sicca syndrome have the same ICD-9 code, “710.2,” we identified “true” SS from CIC holders between 2002 and 2011. These populations were matched with the three cohorts of 1 million random samples from the Longitudinal Health Insurance Database (LHID2000, LHID2005, and LHID2010), which extracted 1 million random samples from 26 million individuals in the NHIRD in 2000, 2005, and 2010. SS subjects were included in this study after removing duplicate data. Figure 1 shows the subject recruitment flowchart from the 3 million random samples for SS with CIC from the NHIRD in Taiwan.

### Data Analysis

SPSS version 19.0 (SPSS Inc., Chicago, IL, USA) used applied to analyze the frequency and patterns of formulae or single herb usage and CHM utilization. Categorical data are presented as absolute numbers and percentages, and continuous data are presented as means ± standard deviations. Data linkage analysis and processing were conducted with Structure Query Language (SQL server 2008, Microsoft Corp., Redmond, WA, USA). In addition, we identified core patterns of CHMs used in treating SS/CIC patients using an open-sourced freeware NodeXL (http://nodexl.codeplex.com/), and all the selected combinations were utilized for network analysis. The line width, ranging from 1 to 10 in the network figure was defined by counts of connections between a certain CHM and co-prescribed CHMs, and thicker widths of line connections indicated crucial prescription patterns. If the line width is thicker between CHM-A and CHM-B than that between CHM-A and CHM-C, the connection between CHM-A and CHM-B is co-prescribed more frequently than CHM-A and CHM-C. The top five core patterns of CHMs were clearly identified within the network analysis of CHM utilization.

## Results

There were 15,914 SS patients with CIC (SS/CIC) in Taiwan from 2002 to 2011; however, there were only 683 SS/CIC with a principal diagnosis of SS in LHID2000, 678 in LHID2005, and 635 in LHID2010, respectively. In these three LHID datasets, more than 90% of SS outpatients had visited TCM clinics. However, not all patients visited TCM clinics for SS, and we only included principal diagnosis SS cases for TCM visits. After removing 23 cases of duplicate data, we found 130 SS patients in LHID2000, 133 in LHID2005, and 126 in LHID2010. There were 366 SS patients and 4,867 visits with a principal diagnosis of SS in TCM clinics from 2002 to 2011. The recruitment flowchart for subjects treated for SS/CIC with CHM from the 3 million random samples in the Taiwan NHIRD is shown in Figure 1.

There were 339 female and 27 male patients with SS/CIC treated with CHM. Female patients preferred using CHM for SS more than male patients did (female:male = 12.56:1). The age-sex-specific frequency of CHM used in SS/CIC patients is illustrated in Table 1, and the age and sex of SS/CIC were recorded only at the time of first visit to TCM clinics. The 50–59 year age group had the highest usage percentage (29.79% and 25.93% for males and females, respectively), and the mean ± SD for age was 54.82 ± 13.00 in females and 57.96 ± 16.42 in males, overall.

The mean ± SD TCM clinic visits among 366 SS/CIC patients was 13.30 ± 20.09. Most SS/CIC patients visited TCM clinics less than 10 times, and the maximum was 129. The mean ± SD medical services provided from 472 TCM doctors was 10.31 ± 19.15. Most TCM doctors treated SS patients less than 10 times, and the maximum was 143. Table 2 shows the SS/CIC visit distribution for patients and doctors in Taiwan.

Table 3 presents the top 10 formulae for treating SS among the 26,733 total CHM prescriptions. The most commonly used formula was “Qi-Ju-Di-Huang-Wan” (9.08%), and the average daily dosage was 4.78 ± 1.62 gram (g). The next most common formulae were “Gan-Lu-Yin” (8.16%), “Jia-Wei-Xiao-Yao-San” (6.02%), “Zhi-Gan-Cao-Tang” (2.06%), and “Xue-Fu-Zhu-Yu-Decoction” (2.03%) with average daily dosages of 4.37 ± 1.78 g, 4.70 ± 1.49 g, 3.65 ± 2.23 g, and 4.50 ± 2.21 g, respectively.

The top 10 single herbs used for SS/CIC are listed in Table 4. “Xuan-Shen” (*Scrophularia ningpoensis* Hemsl., 2.98%) was the most commonly used single herb, and the average daily dosage was 1.86 ± 0.41 g, followed by “Mai-Men-Dong” (*Ophiopogon japonicus* (L. f.) Ker-Gawl., 2.93%), “Sheng-Di-Huang” (raw *Rehmannia glutinosa* Libosch., 21.7%), “Tian-Hua-Fen” (*Trichosanthes kirilowii* Maxim., 2.04%), and “Huang-Qin” (*Scutellaria baicalensis* Georgi, 1.95%) with average daily dosages of 1.38 ± 0.58 g, 1.42 ± 0.52 g, 1.24 ± 0.46 g, and 1.24 ± 0.46 g, respectively.

Supplementary Figure S1 shows that SS/CIC patients were given an average of 6.24 ± 2.47 CHM items in a single prescription, and five CHM items (16.85%) was the most common prescription for combinations of formulae or single herbs. The next most common values were 6 (16.71%) and 7 CHM items (13.77%). Very few SS patients (0.05%) were prescribed more than 20 CHM items.

Among these prescriptions, we analyzed the co-prescription pattern of formulae and single herbs. Supplementary Table S1 shows the most common prescription patterns of two formula combinations and two single herb combinations. The most common two formula combination was “Qi-Ju-Di-Huang-Wan plus Gan-Lu-Yin,” followed by “Jia-Wei-Xiao-Yao-San plus Qi-Ju-Di-Huang-Wan,” “Jia-Wei-Xiao-Yao-San plus Ping-Wei-San,” “Jia-Wei-Xiao-Yao-San plus Zhi-Bo-Di-Huang-Wan,” and “Gan-Lu-Yin plus Sang-Ju-Yin.” The most common two single herb combinations were “Xuan-Shen plus Mai-Men-Dong,” “Sheng-Di-Huang plus Mai-Men-Dong,” “Xuan-Shen plus Sheng-Di-Huang,” “Gou-Qi plus Mai-Men-Dong,” and “Sha-Can (*Glehnia littoralis* F. Schmidt ex Miq.) plus Mai-Men-Dong.”

**Figure 2:**
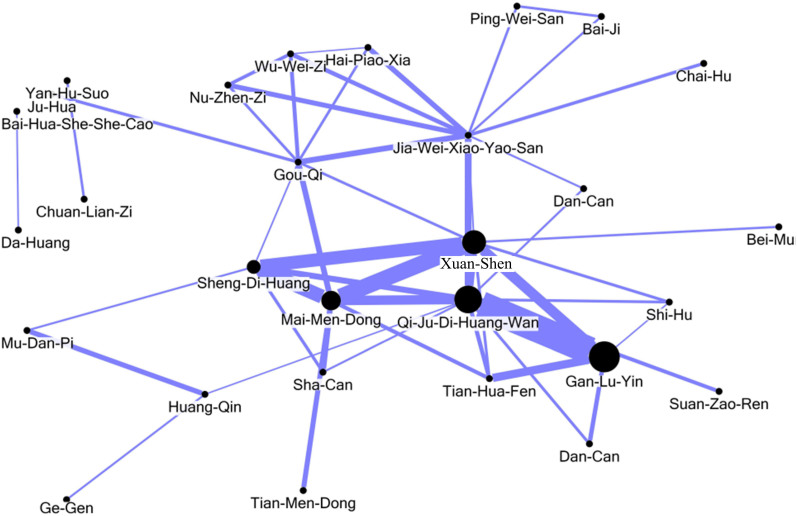
The core pattern of Chinese formula and single herb usage for Sjögren's syndrome. The top 50 Chinese formulae and single herbs for Sjögren's syndrome patients with a catastrophic illness certificate were analyzed through open-sourced freeware NodeXL, and the core pattern of these CHMs showed that Qi-Ju-Di-Huang-Wan, Gan-Lu-Yin, Xuan-Shen, Mai-Men-Dong, and Sheng-Di-Huang are among the most frequently used combinations.

Table 5 shows the most common prescription patterns of double and triple formula/single herb combinations. The most common combination of two formulae/single herbs was “Qi-Ju-Di-Huang-Wan plus Gan-Lu-Yin,” followed by “Xuan-Shen plus Mai-Men-Dong,” “Sheng-Di-Huang plus Mai-Men-Dong,” “Xuan-Shen plus Sheng-Di-Huang,” and “Xuan-Shen and Gan-Lu-Yin.” The most common combination of three formulae/single herbs was “Xuan-Shen, Sheng-Di-Huang, plus Mai-Men-Dong,” followed by “Nu-Zhen-Zi (*Ligustrum lucidum* Ait.), Jia-Wei-Xiao-Yao-San, plus Gou-Qi,” “Nu-Zhen-Zi, Wu-Wei-Zi (*Schizandra chinensis* (Turcz.) Baill.), plus Jia-Wei-Xiao-Yao-San,” “Wu-Wei-Zi, Jia-Wei-Xiao-Yao-San, plus Gou-Qi,” and “Qi-Ju-Di-Huang-Wan, Gan-Lu-Yin, plus Dan-Can.” The network analysis presented in Figure 2 demonstrates that Qi-Ju-Di-Huang-Wan, Gan-Lu-Yin, Xuan-Shen, Mai-Men-Dong, and Sheng-Di-Huang formed the core patterns of Chinese formulae and herbs used to treat SS/CIC patients.

## Discussion

This was the first nationwide population-based survey of the utilization and core pattern analysis of CHM in SS/CIC from the three LHIDs in Taiwan. Female SS/CIC patients used CHM far more frequently than males did (12.56:1 ratio), and females and males aged between 50 and 59 years had the highest rates of CHM use for SS/CIC. This may be accounted for by a lack of active estrogens and androgens, leading to apoptosis in epithelial salivary gland cells^17^. A recent study demonstrated different serum endocrine levels according to SS symptoms, suggesting that oral dryness may be related to low androgen levels, while low estrogen levels manifest as ocular dryness^18^.

In total, there were 15,914 SS/CIC patients from the Taiwan CIC database from 2002 to 2011 (there were 72,391 total cases of generalized autoimmune syndrome requiring lifelong treatment with CIC up to 2012)^19^. There were 366 principal diagnosis SS/CIC patients who used CHM across the three LHIDs with duplicate data removed. The sample size in this study was not large enough, as the LHIDs only provided 1 million random samples, and thus the sample size could not represent all SS/CIC patients undergoing CHM treatment. The database could only represent the ratio of 1 million samples over a total of 23.4 million inhabitants (4.27%). We found that the ratio of SS/CIC patients treated with CHM could be traced back to represent 63.55% of the entire SS/CIC population, a ratio that could also match the nationwide survey of TCM utilization^20^.

Hydroquinone is the main Western medicine used on SS patients in Taiwan; it has immune regulatory effects and can improve fatigue, arthralgia, and myalgia. However, a recent study showed that hydroxychloroquine did not improve symptoms during 24 weeks of treatment for pSS^21^. Saliva and tear substitutes usually treat only dryness symptoms, but some patients received oral corticosteroids, NSAIDs, azathioprine, and cyclosporine for anti-inflammation and immuno-suppression. Pilocarpine and cevimeline are muscarinic acetylcholine receptor agonists for treating dry mouth^22^, but these drugs can cause gastrointestinal discomfort, sweating, flush, and blurred vision. For some SS patients with affected extra-glandular organs, biologic agents, like etanercept and rituximab, might be required to treat severe systemic involvement. However, these are more costly, and they carry many adverse effects. Hence, an increasing number of SS patients seek TCM to treat SS or alleviate the adverse effects of the above drugs.

There were 366 subjects who made TCM clinic visits to 472 TCM doctors; four subjects visited over 100 times from 2002 to 2011, while 238 subjects (65.03%) visited less than 10 times. A similar situation was observed among doctors, such that only three doctors received more than 100 visits, while 343 doctors (72.67%) had been visited less than 10 times. The phenomenon of doctor shopping among patients with chronic illness may account for the observation that many SS/CIC patients received CHM treatment from different TCM doctors^23^. Doctor shopping likely occurred because the health service system of family doctors had not yet been well-established in Taiwan^24^; therefore, patients who have been bothered by persistent symptoms and medication side effects often seek a second opinion^25^. Healthcare resources may be also wasted due to this doctor shopping in TCM^26^.

SS is called “dry-Bi” or “dryness impediment” in TCM, and the TCM pathogenesis of SS is highly correlated with “Yin-deficiency,” manifesting as “dryness-heat” and “consumption and deficiency of qi and body fluid”^27^. Thus, dry eye and dry mouth are considered the external symptoms of the impaired functions. Given this pathogenesis, clinical treatments usually seek to “Enrich yin and clear heat” or “Nourish yin and moisten dryness,” and “Qi-Ju-Di-Huang-Wan,” “Gan-Lu-Yin,” “Xuan-Shen,” and “Mai-Men-Dong” are the commonly used Chinese formulas/single herbs. Some SS patients have fibrotic changes in their salivary glands, which is compatible with the TCM pathogenesis of “Stasis.” “Xue-Fu-Zhu-Yu-Decoction” and “Dan-Can” could “Quicken the blood and dispel stasis.” However, there are some different patterns, like “Qi depression” or “dampness,” for which “Jia-Wei-Xiao-Yao-San” could “Course the liver and resolve depression,” and “Ping-Wei-San” could “Dry dampness and fortify the spleen.” TCM doctors usually recognize the TCM patterns of SS first, and then prescribe different formula or single herbs accordingly.

“Qi-Ju-Di-Huang-Wan” was the most commonly used formula for SS/CIC; its therapeutic function is to “nourish the liver and brighten the eyes,” and it could be used for SS and dry eye. Recent studies have reported that “Qi-Ju-Di-Huang-Wan” is more effective than conventional medicine for SS, regardless of whether it is used alone^28^ or in addition to acupuncture^29^. Chang^30^ used “Qi-Ju-Di-Huang-Wan” with a randomized, double-masked, parallel grouped controlled study to treat dry eyes, and the result showed reduction in corneal epithelium abnormalities, and it could also act as an alternative to topical eye drops. However, subjects in Chang's study had dry eye syndrome, not SS, and thus “Qi-Ju-Di-Huang-Wan” may have therapeutic effects for dry eye symptoms of SS.

“Gan-Lu-Yin” was the second most commonly used formula in our database; its therapeutic function is to “enrich yin and clear heat,” and it can be used for the treatment of SS and dry mouth. A study used “Gan-Lu-Yin” in post-radiotherapy nasopharyngeal cancer patients to alleviate mucositis and dry mouth^31^. “Jia-Wei-Xiao-Yao-San” can “course the liver and resolve depression,” and Yi et al.^32^ used it to treat xerophthalmia in perimenopausal women with significant improvement of visual fatigue sensation, red eye, dryness, foreign body sensation, burning sensation, and photophobia. “Zhi-Gan-Cao-Tang” can “boost qi and enrich yin”; however, no studies have discussed it for treating SS-related symptoms. “Xue-Fu-Zhu-Yu-Decoction” can “quicken the blood and dispel stasis,” and it can modulate the immune function of B cells and T cells^33^ and alleviate tissue fibrosis^34^.

“Xuan-Shen” was the most commonly used single herb, with the function of “enrich yin and clear heat,” and it has some positive effects on anti-inflammation and tumor cell apoptosis^35^. “Mai-Men-Dong” was the second most commonly used single herb to “nourish yin and moisten dryness”; it can down-regulate mRNA expression of TGF-β1 and has potential antioxidant effects^36^. “Sheng-Di-Huang” can “enrich yin and clear heat,” and it also reduces eosinophil cationic proteins levels, which are positively correlated with “heat zheng”^37^ and has anti-inflammatory properties^38^. “Tian-Hua-Fen” can “clear heat and engender liquid,” and it can inhibit nitric oxide activity and elevate anti-inflammatory effects^39^. “Huang-Qin” can “clear heat and drain fire,” and it has the function of scavenging reactive oxygen species^40^. We also listed relevant studies for the top 10 most commonly used formulae and single herbs in Tables 3 and 4.

Figure 2 includes the top 50 Chinese formulas and single herbs for SS/CIC patients and shows the core pattern of these CHMs. This figure implies that “Qi-Ju-Di-Huang-Wan,” “Gan-Lu-Yin, Xuan-Shen,” “Mai-Men-Dong,” and “Sheng-Di-Huang” are among the most frequently used combinations. Although these items may have effects on antioxidant capacity, anti-inflammation, and dry eye or dry month improvement, they have rarely been applied in clinical trials. Furthermore, these five CHMs from the core pattern identified did little have immune modulation or tissue fibrosis alleviation functions. The above CHMs may have effects on immune regulation, but there is little evidence to support this point. This kind of explanation according to modern biomedical categories may be biased and downplay TCM theory and CHM use. There may indeed be undiscovered immune regulation effects, so we are now conducting a clinical trial attempting to determine the mechanism of SS-1 and its components with antioxidant capacity, anti-inflammation, immune modulation, and anti-fibrosis cell-line basic research. In the future, we may further use this model to screen other CHMs for treating SS. However, no specific CHM could be recommended for clinical use^11^.

For this purpose, we initiated a randomized, double-blind, placebo-controlled, cross-over design clinical trial (Clinicaltrials.gov NCT02110446) for SS/CIC patients in order to evaluate the efficacy of CHM (SS-1) on the regulation of oxidative stress-related cytokines and antioxidant capacity. “Gan-Lu-Yin, Sang-Ju-Yin, and Xue-Fu-Zhu-Yu-Decoction” with a ratio of 2:1:1 is the composition of SS-1, and its therapeutic functions include antioxidant capacity, anti-inflammation, immune modulation, anti-fibrosis, and dry mouth improvement^31^,^33^,^34^,^41^,^42^. This combination of SS-1 has been effective in our clinical experience. We use “Gan-Lu-Yin” for “Enrich yin and clear heat,” “Xue-Fu-Zhu-Yu-Decoction,” for “quicken the blood and dispel stasis,” and “Sang-Ju-Yin” for “Course wind and discharge heat.” Thus, SS-1 can improve dryness symptoms of dry eye, dry mouth, and other exocrine glands. As the core patterns in our database were treated the patient with “enrich yin” most frequently, and “Qi-Ju-Di-Huang-Wan” takes long time to enrich the internal yin (liver yin and kidney yin), before spreading to the exterior. Thus, we use “Gan-Lu-Yin” to enrich the exterior yin first, and “Sang-Ju-Yin” can bring these fluids and yin outside to superficial areas for the mucosa of exocrine glands. However, some patients have had SS for a long time, and the salivary glands come to a state of “Qi stagnation and blood stasis.” “Xue-Fu-Zhu-Yu-Decoction” can improve this situation. Although SS-1 is frequently used in our clinical practice for treating with SS, it was not yet have evidence-based support by a well-designed RCT to verify its efficacy and safety. Thus, we initiated a well-designed clinical trial and we expect improvements in quality of life and clinical manifestations through reduction of oxidative stress. We also plan to use an SS cell model to elucidate the antioxidant effects and the mechanism of action of SS-1.

As this study was a retrospective study, we could not recognize the different herbs or formulae that were selected for different presentations of SS according to TCM diagnosis. This is indeed the limitation of our study, but ours results could still demonstrate overall patterns in TCM treatment for SS. We might develop further studies to discuss the clinical thinking processes among TCM doctors. In our ongoing SS-1 clinical trial, we also want to observe whether the uniform treatment for the various patterns of SS-1 lead to different outcomes.

Years of practicing TCM experience may affect clinical treatment decision making - this is an important issue and a potential confounding factor. However, the NHIRD did not capture the information about patients' TCM patterns, years of practicing TCM experience, and the practitioners' education, so we could not analyze these factors in our study. However, it deserves to conduct the survey for these TCM variables in the future.

Our analysis shows different results from a recent SS report surveying CHM prescription patterns in Taiwan from 1997 to 2008^13^. (1) We included SS/CIC subjects from three LHIDs between 2002 and 2011 for a comprehensive and up-to-date nationwide survey. (2) We only included principal diagnosis of SS/CIC in TCM visits to reduce the measurement bias due to TCM visits for non-SS treatments. (3) The statistics of SS/CIC patient numbers and the dosage of the top 10 Chinese formulae demonstrated more rational and significant results in this study.

However, there are some limitations to this study. (1) Many potential SS patients accepted conventional therapy or TCM, but they were not CIC holders due to incompatibility of histopathology or autoantibody criteria. As the SS subjects included in this study were required to have a CIC, the CHM utilization among SS patients may have been underestimated. (2) The sample size in this study was not large enough; hence, we should conduct another larger survey on this issue in the future. (3) As the subjects included in this study were extracted from three LHIDs, multivariate logistic regression was not conducted to evaluate factors correlated with CHM utilization. (4) We studied only the utilization of CHM in the recruited SS/CIC patients; however, we did not investigate the utilization of acupuncture, Chinese tuina, or other CAMs, which might also be applied in SS therapies. (5) The safety data in this retrospective study is lacking, and so we cannot evaluate the safety of CHM.

## Conclusions

“Qi-Ju-Di-Huang-Wan” was the most commonly used formula, while “Xuan-Shen” was the most commonly used single herb in our database. Among different age groups, the highest utilization of CHM was found between ages 50 and 59 in both females and males. The most commonly used two formula combination was “Qi-Ju-Di-Huang-Wan plus Gan-Lu-Yin,” and the most commonly used two single herb combination was “Xuan-Shen plus Mai-Men-Dong.” The core pattern prescriptions were “Qi-Ju-Di-Huang-Wan,” “Gan-Lu-Yin,” “Xuan-Shen,” “Sheng-Di-Huang,” and “Mai-Men-Dong.” However, the therapeutic effects and safety of these commonly used CHMs for treating SS have not been clearly elaborated, so well-designed clinical trials for this purpose are required in the future.

## Supplementary Material

Supplementary Information Supplementary Information

## Figures and Tables

**Table 1:** Age distribution of Sjögren's syndrome patients with catastrophic illness certificate treated with Chinese herbal medicine

	Total	Female	Male	Female/Male Ratio
Number	366	339	27	12.56
Age				
< 20	1 (0.27%)	1 (0.29%)	0 (0.00%)	-
20–29	9 (2.46%)	7 (2.06%)	2 (7.41%)	3.5
30–39	38 (10.38%)	36 (10.62%)	2 (7.41%)	18
40–49	72 (19.67%)	68 (20.06%)	4 (14.81%)	17
50–59	108 (29.51%)	101 (29.79%)	7 (25.93%)	14.43
60–69	86 (23.50%)	81 (23.89%)	5 (18.52%)	16.2
70–79	40 (10.93%)	36 (10.62%)	4 (14.81%)	9
≧ 80	12 (3.28%)	9 (2.65%)	3 (11.11%)	3
Mean ± SD	55.05 ± 13.28	54.82 ± 13.00	57.96 ± 16.42	
Max	90	90	85	
Min	16	16	28	
Range	74	74	57	

**Table 2:** Visit distribution of patients and doctors of Sjögren's syndrome with catastrophic illness certificate treated with Chinese herbal medicine

	Patient	Doctor
Number	366	472
Total	4867	4867
Visit		
< 10	238 (65.03%)	343 (72.67%)
10–19	55 (15.03%)	66 (13.98%)
20–29	19 (5.19%)	26 (5.51%)
30–39	19 (5.19%)	9 (1.91%)
40–49	10 (2.73%)	6 (1.27%)
50–59	11 (3.01%)	5 (1.06%)
60–69	1 (0.27%)	2 (0.42%)
70–79	5 (1.37%)	2 (0.42%)
80–89	3 (0.82%)	4 (0.85%)
90–99	1 (0.27%)	6 (1.27%)
100–109	2 (0.55%)	1 (0.21%)
110–119	1 (0.27%)	0 (0.00%)
120–129	1 (0.27%)	0 (0.00%)
130–139	0 (0.00%)	0 (0.00%)
≧ 140	0 (0.00%)	2 (0.42%)
Mean ± SD	13.30 ± 20.09	10.31 ± 19.15
Max	129	143
Min	1	1
Range	128	142

**Table 3:** The top 10 formula of Sjögren's syndrome with catastrophic illness certificate in Taiwan (total prescription numbers = 26,733)

	TCM prescription	Ingredients	Therapeutic actions and indications^43^	Frequency of prescription N (%)	Average daily dosage (g) (Mean ± SD)
1	Qi-Ju-Di-Huang-Wan	Ju-Hua (*Chrysanthemum morifolium* (Ramat.) Tzvel.), Gou-Qi (*Lycium barbarum* L.), Shou-Di-Huang (*Rehmannia glutinosa* Libosch.), Shan-Zhu-Yu (*Cornus officinalis* Sieb. et Zucc.), Shan-Yao (*Dioscorea opposita* Thunb.), Ze-Xie (*Alisma orientalis* (Sam.) Juzep.), Fu-Ling (*Poria cocos* (Schw.) Wolff), Mu-Dan-Pi (*Paeonia suffruticosa* Andr.)	Nourish the liver and brighten the eyes E^28^,^29^,^30^	834 (9.08%)	4.78 ± 1.62
2	Gan-Lu-Yin	Sheng-Di-Huang (raw *Rehmannia glutinosa* Libosch.), Shou-Di-Huang (*Rehmannia glutinosa* Libosch.), Tian-Men-Dong (*Asparagus cochinchinensis* (Lour.) Merr.), Mai-Men-Dong (*Ophiopogon japonicus* (L. f.) Ker-Gawl.), Shi-Hu (*Dendrobium chrysanthum* Wall.), Yin-Chen-Hao (*Artemisia capillaris* Thunb.), Huang-Qin (*Scutellaria baicalensis* Georgi), Zhi-Ke (*Citrus aurantium* L.), Pi-Pa-Ye (*Eriobotrya japonica* (Thunb.) Lindl.), Zhi-Gan-Cao (*Glycyrrhiza glabra* L.)	Enrich yin and clear heat A^31^	750 (8.16%)	4.37 ± 1.78
3	Jia-Wei-Xiao-Yao-San	Zhi-Gan-Cao (*Glycyrrhiza glabra* L.), Dang-Gui (*Angelica sinensis* (Oliv.) Diels), Fu-Ling (*Poria cocos* (Schw.) Wolff), Bai-Shao-Yao (*Paeonia lactiflora* Pall.), Bai-Zhu (*Atractylodes macrocephala* Koidz.), Chai-Hu (*Bupleurum chinense* DC.), Sheng-Jiang (*Zingiber officinale* Rosc.), Bo-He (*Mentha haplocalyx* Briq.), Mu-Dan-Pi (*Paeonia suffruticosa* Andr.), Zhi-Zi (*Gardenia jasminoides* Ellis)	Course the liver and resolve depression E^32^	553 (6.02%)	4.70 ± 1.49
4	Zhi-Gan-Cao-Tang	Zhi-Gan-Cao (*Glycyrrhiza glabra* L.), Sheng-Jiang (*Zingiber officinale* Rosc.), Ren-Can (*Panax ginseng* C. A. Meyer), Sheng-Di-Huang (raw *Rehmannia glutinosa* Libosch.), Gui-Zhi (*Cinnamomun cassia* Presl), A-Jiao (*Equus asinus* L.), Mai-Men-Dong (*Ophiopogon japonicus* (L. f.) Ker-Gawl.), Ma-Zi-Ren (*Cannabis sativa* L.), Da-Zao (*Ziziphus jujuba* Mill.)	Boost qi and enrich yin	189 (2.06%)	3.65 ± 2.23
5	Xue-Fu-Zhu-Yu-Decoction	Dang-Gui (*Angelica sinensis* (Oliv.) Diels), Sheng-Di-Huang (raw *Rehmannia glutinosa* Libosch.), Tao-Ren (*Prunus persica* (L.) Batsch), Hong-Hua (*Carthamus tinctorius* L.), Zhi-Ke (*Citrus aurantium* L.), Chi-Shao (red *Paeonia lactiflora* Pall.), Chai-Hu (*Bupleurum chinense* DC.), Zhi-Gan-Cao (*Glycyrrhiza glabra* L.), Jie-Geng (*Platycodon grandiflorum* (Jacq.) A. DC.), Chuan-Qiong (*Ligusticum chuanxiong* Hortorum), Niu-Xi (*Achyranthes bidentata* Blume)	Quicken the blood and dispel stasis C and D^33^,^34^	187 (2.03%)	4.50 ± 2.21
6	Yi-Guan-Jian	Dang-Gui (*Angelica sinensis* (Oliv.) Diels), Bai-Zhu (*Atractylodes macrocephala* Koidz.), Fu-Ling (*Poria cocos* (Schw.) Wolff), Gou-Teng (*Uncaria rhynchophylla* (Miq.) Jacks.), Chuan-Qiong (*Ligusticum chuanxiong* Hortorum), Chai-Hu (*Bupleurum chinense* DC.), Zhi-Gan-Cao (*Glycyrrhiza glabra* L.)	Coursing the liver and fortify the spleen A and D^44^,^45^,^46^,^47^,^48^	185 (2.01%)	3.58 ± 1.44
7	Zhi-Bo-Di-Huang-Wan	Zhi-Mu (*Anemarrhena asphodeloides* Bunge), Huang-Bo (*Phellodendron chinense* Schneid.), Shou-Di-Huang (*Rehmannia glutinosa* Libosch.), Shan-Zhu-Yu (*Cornus officinalis* Sieb. et Zucc.), Shan-Yao (*Dioscorea opposita* Thunb.), Ze-Xie (*Alisma orientalis* (Sam.) Juzep.), Fu-Ling (*Poria cocos* (Schw.) Wolff), Mu-Dan-Pi (*Paeonia suffruticosa* Andr.)	Enrich yin and clear heat E^49^	183 (1.99%)	4.27 ± 1.56
8	Ping-Wei-San	Cang-Zhu (*Atractylodes lancea* (Thunb.) DC.), Hou-Po (*Magnolia officinalis* Rehd. et Wils.), Chen-Pi (*Citrus reticulata* Blanco), Zhi-Gan-Cao (*Glycyrrhiza glabra* L.)	Dry dampness and fortify the spleen	183 (1.99%)	2.56 ± 1.09
9	Sha-Can-Mai-Dong-Tang	Sha-Can (*Glehnia littoralis* F. Schmidt ex Miq.), Yu-Zhu (*Polygonatum odoratum* (Mill.) Druce), Sheng-Gan-Cao (raw *Glycyrrhiza glabra* L.), Sang-Ye (*Morus alba* L.), Mai-Men-Dong (*Ophiopogon japonicus* (L. f.) Ker-Gawl.), Bian-Dou (*Dolichos lablab* L.), Tian-Hua-Fen (*Trichosanthes kirilowii* Maxim*.*)	Nourish yin and engender liquid C^50^	174 (1.89%)	4.31 ± 1.93
10	Ma-Zi-Ren-Wan	Ma-Zi-Ren (*Cannabis sativa* L.), Bai-Shao-Yao (*Paeonia lactiflora* Pall.), Zhi-Shi (*Citrus aurantium* L.), Da-Huang (*Rheum tanguticum* Maxim. ex Balf.), Hou-Po (*Magnolia officinalis* Rehd. et Wils.), Xing-Ren (*Prunus armeniaca* L.)	Moisten the intestines and drain fire	165 (1.80%)	2.78 ± 1.59
29	Sang-Ju-Yin	Sang-Ye (*Morus alba* L.), Ju-Hua (*Chrysanthemum morifolium* (Ramat.) Tzvel.), Lian-Qiao (*Forsythia suspense* (Thunb.) Vahl), Bo-He (*Mentha haplocalyx* Briq.), Jie-Geng (*Platycodon grandiflorum* (Jacq.) A. DC.), Zhi-Gan-Cao (*Glycyrrhiza glabra* L.), Lu-Gen (*Phragmites communis* Trinus)	Course wind and discharge heat B and C^41^,^42^	89 (0.97%)	3.75 ± 1.26

A: antioxidant capacity, B: anti-inflammation, C: immune modulation, D: anti-fibrosis, E: improve dry eye or dry month

**Table 4:** The top 10 single herbs of Sjögren's syndrome with catastrophic illness certificate in Taiwan (total prescription numbers = 26,733)

TCM prescription	Ingredients	Therapeutic actions and indications^51^	Frequency of prescription N (%)	Average daily dosage (g) (Mean ± SD)
Xuan-Shen	*Scrophularia ningpoensis* Hemsl.	Enrich yin and clear heat B^35^	(2.98%)	1.86 ± 0.41
Mai-Men-Dong	*Ophiopogon japonicus* (L. f.) Ker-Gawl.	Nourish yin and moisten dryness A^36^	514 (2.93%)	1.38 ± 0.58
Sheng-Di-Huang	raw *Rehmannia glutinosa* Libosch*.*	Enrich yin and clear heat B^38^	380 (2.17%)	1.42 ± 0.52
Tian-Hua-Fen	*Trichosanthes kirilowii* Maxim*.*	Clear heat and engender liquid A and B^39^	357 (2.04%)	1.24 ± 0.46
Huang-Qin	*Scutellaria baicalensis* Georgi	Clear heat and drain fire A^40^	342 (1.95%)	1.24 ± 0.46
Gou-Qi	*Lycium barbarum* L.	Boost essence and brighten the eyes A and B^52^,^53^	322 (1.84%)	1.27 ± 0.59
Da-Huang	*Rheum tanguticum* Maxim. ex Balf.	Clear heat and drain fire A, B and C^54^,^55^,^56^,^57^,^58^,^59^	294 (1.68%)	1.07 ± 0.73
Ju-Hua	*Chrysanthemum morifolium* (Ramat.) Tzvel.	Course wind and discharge heat A^60^	292 (1.66%)	1.18 ± 0.38
Dan-Can	*Salvia miltiorrhiza* Bge.	Cool the blood and clear heat A and C^61^,^62^,^63^	289 (1.65%)	1.20 ± 0.44
Shi-Hu	*Dendrobium chrysanthum* Wall.	Enrich yin and clear heat A and B^64^	282 (1.61%)	1.23 ± 0.46

A: antioxidant capacity, B: anti-inflammation, C: immune modulation, D: anti-fibrosis

**Table 5:** The most common prescription patterns for two and triple drugs combination in a single prescription of Sjögren's syndrome with catastrophic illness certificate

	Name			Number of prescriptions N (%)
**Two combination**				
1	Qi-Ju-Di-Huang-Wan	Gan-Lu-Yin		268 (0.322%)
2	Xuan-Shen	Mai-Men-Dong		176 (0.212%)
3	Sheng-Di-Huang	Mai-Men-Dong		160 (0.193%)
4	Xuan-Shen	Sheng-Di-Huang		152 (0.183%)
5	Xuan-Shen	Gan-Lu-Yin		130 (0.156%)
6	Qi-Ju-Di-Huang-Wan	Mai-Men-Dong		123 (0.148%)
7	Tian-Hua-Fen	Gan-Lu-Yin		113 (0.136%)
8	Xuan-Shen	Qi-Ju-Di-Huang-Wan		110 (0.132%)
9	Jia-Wei-Xiao-Yao-San	Qi-Ju-Di-Huang-Wan		102 (0.123%)
10	Sheng-Di-Huang	Qi-Ju-Di-Huang-Wan		95 (0.114%)
**Triple combination**				
1	Xuan-Shen	Sheng-Di-Huang	Mai-Men-Dong	96 (0.055%)
2	Nu-Zhen-Zi	Jia-Wei-Xiao-Yao-San	Gou-Qi	66 (0.038%)
3	Nu-Zhen-Zi	Wu-Wei-Zi	Jia-Wei-Xiao-Yao-San	66 (0.038%)
4	Wu-Wei-Zi	Jia-Wei-Xiao-Yao-San	Gou-Qi	64 (0.037%)
5	Qi-Ju-Di-Huang-Wan	Gan-Lu-Yin	Dan-Can	63 (0.036%)
6	Nu-Zhen-Zi	Wu-Wei-Zi	Gou-Qi	59 (0.034%)
7	Jia-Wei-Xiao-Yao-San	Ping-Wei-San	Bai-Ji	52 (0.030%)
8	Da-Huang	Bai-Hua-She-She-Cao	Mei-Yao	49 (0.028%)
9	Jia-Wei-Xiao-Yao-San	Gou-Qi	Hai-Piao-Xiao	47 (0.027%)
10	Xuan-Shen	Gan-Lu-Yin	Qi-Ju-Di-Huang-Wan	47 (0.027%)

Bai-Ji****: *Bletilla striata* (Thunb.) Rechib. f., Mei-Yao****: *Commiphora myrrha* Engler, Hai-Piao-Xiao: *Sepia esculenta* Hoyle.
