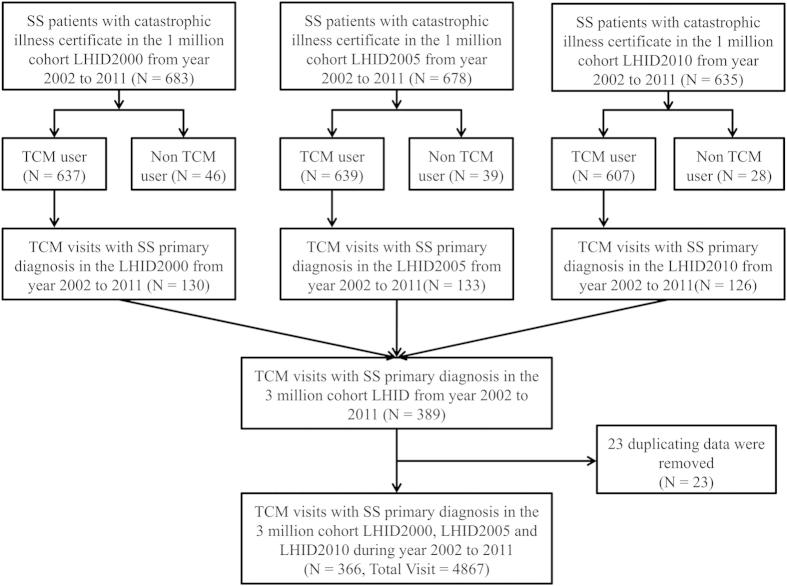# Corrigendum: The Core Pattern Analysis on Chinese Herbal Medicine for Sjögren’s syndrome: A Nationwide Population-Based Study

**DOI:** 10.1038/srep14887

**Published:** 2015-10-08

**Authors:** Ching-Mao Chang, Hsueh-Ting Chu, Yau-Huei Wei, Fang-Pey Chen, Shengwen Wang, Po-Chang Wu, Hung-Rong Yen, Tzeng-Ji Chen, Hen-Hong Chang

Scientific Reports 5: Article number: 9541; 10.1038/srep09541 published online: 04292015; updated: 10082015.

This Article contains typographical errors in the flowchart of Fig. 1. The correct Fig. 1 appears below.

In addition, there are typographical errors in the Methods section under subheading ‘Identification of Patients with Sjögren’s Syndrome’:

“These populations were matched with the three cohorts of 1 million random samples from the Longitudinal Health Insurance Database (LHID2000, LHID2005, and LHID2010), which extracted 1 million random samples from 26 million individuals in the NHIRD in 2000, 2005, and 2010.”

should read:

“These populations were matched with the three cohorts of 1 million random samples from the Longitudinal Health Insurance Database (LHID2000, LHID2005, and LHID2010), which extracted 1 million random samples from 23.4 million individuals in the NHIRD in 2000, 2005, and 2010.”

In the Results section:

“Sheng-Di-Huang” (raw *Rehmannia glutinosa* Libosch., 21.7%)”

should read:

“Sheng-Di-Huang” (raw *Rehmannia glutinosa* Libosch., 2.17%)”

And lastly, in Table 4, the ‘Frequency of prescription N (%)’ for Xuan-Shen ‘(2.98%)’ should read ‘522 (2.98%).’

## Figures and Tables

**Figure 1:**