# Corrigendum: The Core Pattern Analysis on Chinese Herbal Medicine for Sjögren’s syndrome: A Nationwide Population-Based Study

**DOI:** 10.1038/srep46824

**Published:** 2017-05-25

**Authors:** Ching-Mao Chang, Hsueh-Ting Chu, Yau-Huei Wei, Fang-Pey Chen, Shengwen Wang, Po-Chang Wu, Hung-Rong Yen, Tzeng-Ji Chen, Hen-Hong Chang

Scientific Reports 5: Article number: 9541; 10.1038/srep09541 published online: 04 29 2015; updated: 05 25 2017.

In this Article, Affiliation 2 is incorrectly listed as ‘Graduate Institute of Clinical Medicine, and Graduate Institute of Traditional Chinese Medicine, College of Medicine, Chang Gung University, Taoyuan, Taiwan’. The correct affiliation is listed below:

Graduate Institute of Clinical Medical Sciences, College of Medicine, Chang Gung University, Taoyuan, Taiwan.

