# Supplementary material for: The Core Pattern Analysis on Chinese Herbal Medicine for Sjögren's syndrome: A Nationwide Population-Based Study
Source: Sci Rep. 2015 Apr 29;5:9541. doi: 10.1038/srep09541 (PMC5386216; doi:10.1038/srep09541)
Supplement: Supplementary Information [file srep09541-s1.pdf]

Supplement

The Core Pattern Analysis on Chinese Herbal Medicine for Sjögren's syndrome: A Nationwide Population-Based Study

Ching-Mao Chang [1,2], Hsueh-Ting Chu [3,4,9], Yau-Huei Wei [5,6], Fang-Pey Chen [2], Shengwen Wang [7], Po-Chang Wu [8], Hung-Rong Yen [9], Tzeng-Ji Chen [a,b,*], Hen-Hong Chang [9,*]

[*] Correspondence and co-orrespondence to: Prof. Hen-Hong Chang and Prof. Tzeng-Ji Chen

Prof. Hen-Hong Chang
Research Center for Chinese Medicine & Acupuncture,
China Medical University
No.91, Hsueh-Shih Road, North District, Taichung 40402, Taiwan, R.O.C
Tel: +886-4-22053366 #3126
Fax: +886-4-22037690
E-mail: tcmchh55@gmail.com

Prof. Tzeng-Ji Chen
Department of Family Medicine,
Taipei Veterans General Hospital
No. 201, Sec. 2, Shih-Pai Road, Beitou District, Taipei 112, Taiwan, R.O.C.
Tel: 886-2-28757460
Fax: 886-2-28737901
Email: tjchen@vghtpe.gov.tw

Supplementary Table S1 | The most common prescription patterns for two formulae and two single herbs combination in a single prescription of Sjögren's syndrome patients with catastrophic illness certificate in Taiwan

	Name		Number of prescriptions N (%)
**Two formulae combination**			
1	Qi-Ju-Di-Huang-Wan	Gan-Lu-Yin	268 (3.34%)
2	Jia-Wei-Xiao-Yao-San	Qi-Ju-Di-Huang-Wan	102 (1.27%)
3	Jia-Wei-Xiao-Yao-San	Ping-Wei-San	68 (0.85%)
4	Jia-Wei-Xiao-Yao-San	Zhi-Bo-Di-Huang-Wan	57 (0.71%)
5	Gan-Lu-Yin	Sang-Ju-Yin	56 (0.70%)
6	Jia-Wei-Xiao-Yao-San	Gan-Lu-Yin	55 (0.69%)
7	Qi-Ju-Di-Huang-Wan	Shen-Tong-Zhu-Yu-Tang	53 (0.66%)
8	Jia-Wei-Xiao-Yao-San	Huang-Lian-Jie-Du-Tang	51 (0.64%)
9	Jia-Wei-Xiao-Yao-San	Ma-Zi-Ren-Wan	49 (0.61%)
10	Qi-Ju-Di-Huang-Wan	Xin-Yi-Qing-Fei-Tang	42 (0.52%)
**Two single herbs combination**			
1	Xuan-Shen	Mai-Men-Dong	176 (0.41%)
2	Sheng-Di-Huang	Mai-Men-Dong	160 (0.37%)
3	Xuan-Shen	Sheng-Di-Huang	152 (0.35%)
4	Gou-Qi	Mai-Men-Dong	93 (0.22%)
5	Sha-Can	Mai-Men-Dong	92 (0.21%)
6	Tian-Men-Dong	Mai-Men-Dong	88 (0.20%)
7	Mu-Dan-Pi	Huang-Qin	88 (0.20%)
8	Wu-Wei-Zi	Gou-Qi	79 (0.18%)
9	Tian-Hua-Fen	Mai-Men-Dong	79 (0.18%)
10	Nu-Zhen-Zi	Wu-Wei-Zi	75 (0.17%)

[#] Nu-Zhen-Zi: *Ligustrum lucidum* Ait., Wu-Wei-Zi: *Schizandra chinensis* (Turcz.) Baill.

Among these prescriptions, we evaluated the co-prescription pattern of formula and single herb. Supplementary Table S1 shows the most common prescription patterns of two formulae combination and two single herbs combination. The most common item of two formulae combination was “Gan-Lu-Yin plus Qi-Ju-Di-Huang-Wan”, followed by “Jia-Wei-Xiao-Yao-San plus Qi-Ju-Di-Huang-Wan”, “Jia-Wei-Xiao-Yao-San plus Ping-Wei-San”, “Jia-Wei-Xiao-Yao-San plus Zhi-Bo-Di-Huang-Wan” and “Gan-Lu-Yin plus Sang-Ju-Yin.” And the most common items of two single herbs were “Xuan-Shen plus Mai-Men-Dong”, “Sheng-Di-Huang plus Mai-Men-Dong”, “Xuan-Shen plus Sheng-Di-Huang”, “Gou-Qi plus Mai-Men-Dong” and “Sha-Can plus Mai-Men-Dong.”

Figure Legends

Supplementary Figure S1 | Relationship between the numbers of Chinese formula and single herbs for the Sjögren's syndrome patients. Sjögren's syndrome patients with catastrophic illness certificate was given an average item of 6.24 ± 2.47 CHMs in a single prescription, and five items of CHMs (16.85%) was the most common prescription with the combination of formulae or single herbs.

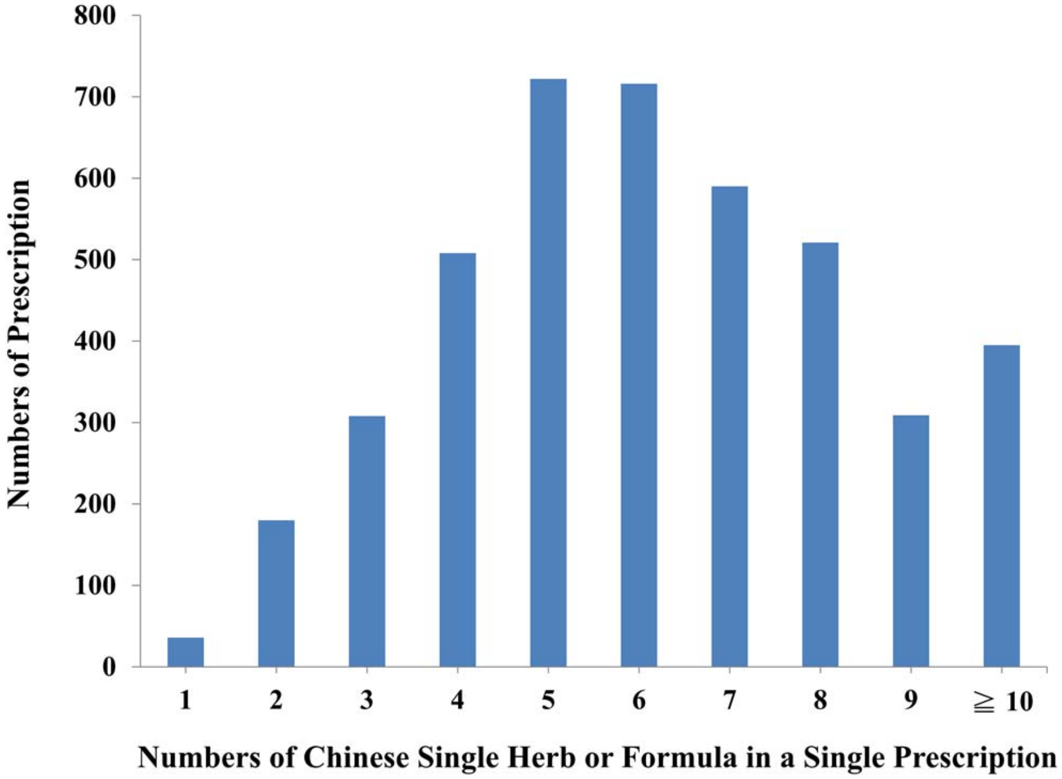

Supplementary Figure S1 | Relationship between the numbers of Chinese formula and single herbs for the Sjögren's syndrome patients. Sjögren's syndrome patients with catastrophic illness certificate was given an average item of 6.24 ± 2.47 CHMs in a single prescription, and five items of CHMs (16.85%) was the most common prescription with the combination of formulae or single herbs.

Supplementary Figure S1 shows that a SS/CIC patient was given an average item of 6.24 ± 2.47 CHM in a single prescription, and five items of CHM (16.85%) was the most common CHM prescription with the combination of formula or single herbs. The following ones were 6 CHM (16.71%) and 7 CHM (13.77%). Some SS patients, only at very low ratios (0.05%), were prescribed more than 20 CHM.
